# Comparative Phenotypic and PCR-Based Methicillin Resistance Characterization of Clinical Canine *Staphylococcus pseudintermedius*

**DOI:** 10.3390/antibiotics15050473

**Published:** 2026-05-07

**Authors:** Patrik Mag, Enikő Fehér, Eszter Kaszab, Dóra Máté, Noémi Tarpataki, Ákos Jerzsele

**Affiliations:** 1 Department of Pharmacology and Toxicology, University of Veterinary Medicine Budapest, H-1078 Budapest, Hungary; 2 National Laboratory of Infectious Animal Diseases, Antimicrobial Resistance, Veterinary Public Health and Food Chain Safety, University of Veterinary Medicine Budapest, H-1078 Budapest, Hungary; 3 Department of Microbiology and Infectious Diseases, University of Veterinary Medicine Budapest, H-1078 Budapest, Hungary; 4 National Laboratory of Virology, Szentágothai Research Centre, University of Pécs, H-7624 Pécs, Hungary; 5 Department of Bioinformatics, One Health Institute, Faculty of Health Sciences, University of Debrecen, H-4032 Debrecen, Hungary; 6 Department of Internal Medicine, University of Veterinary Medicine Budapest, H-1078 Budapest, Hungary

**Keywords:** *Staphylococcus pseudintermedius*, antimicrobial resistance, methicillin-resistant *Staphylococcus pseudintermedius*, multidrug resistance, canine, skin infections, minimum inhibitory concentration, *mecA*, antimicrobial susceptibility testing

## Abstract

**Background:** Antimicrobial resistance in *Staphylococcus pseudintermedius* is an increasing concern in small animal medicine, particularly due to methicillin-resistant strains and associated multidrug resistance. This study aimed to characterize antimicrobial susceptibility patterns in clinical canine isolates, determine the prevalence of methicillin resistance, and assess the presence of *mecA* and *mecC* genes. **Methods:** A total of 243 clinical isolates from canine skin samples collected in Hungary between 2023 and 2025 were analyzed. Minimum inhibitory concentrations (MICs) for 24 antimicrobial agents were determined using broth microdilution in accordance with CLSI guidelines. Isolates were classified as methicillin-resistant or susceptible based on oxacillin MIC values. PCR assays targeting *mecA* and *mecC* were performed on oxacillin-resistant isolates. **Results:** Of the 243 isolates, 47 (19.3%) were methicillin-resistant. High resistance rates were observed for β-lactams, tetracyclines, macrolides, and lincosamides, while rifampicin, amikacin and florfenicol retained good to excellent activity. Methicillin-resistant isolates exhibited substantially higher resistance across multiple antimicrobial classes. Overall, 50.6% of all isolates were classified as multidrug-resistant (MDR) and 17.3% as extensively drug-resistant (XDR), while no pandrug-resistant (PDR) isolates were detected; all methicillin-resistant isolates were at least MDR. The *mecA* gene was detected in 80.9% of oxacillin-resistant isolates, while *mecC* was not identified. **Conclusions:** Methicillin resistance in canine *S. pseudintermedius* is closely associated with multidrug resistance, limiting therapeutic options. However, selected agents, including rifampicin, amikacin and florfenicol, retained in vitro activity. Partial discordance between phenotypic resistance and *mecA* detection highlights the importance of combined phenotypic and molecular approaches. These findings have direct implications for empirical antimicrobial therapy and support targeted treatment strategies, while emphasizing the importance of antimicrobial stewardship in small animal practice.

## 1. Introduction

Antimicrobial resistance (AMR) is one of the most serious challenges to global health, affecting both human and veterinary medicine. The One Health approach highlights the interconnected nature of human, animal and environmental health, including the circulation of resistant microorganisms and resistance genes between these systems [1]. In addition to food-producing animals, companion animals—particularly dogs—play a significant role, as they live in close contact with humans, often within the same household. This proximity facilitates the transmission of resistant bacteria between animals and humans [2,3]. The zoonotic potential of methicillin-resistant staphylococci has been demonstrated in several studies, particularly among individuals in intensive contact with dogs [4,5,6].

In companion animals, one of the most common indications for antimicrobial therapy is bacterial skin and soft tissue infections. In dogs, dermatological diseases—particularly bacterial pyoderma—are among the leading causes of veterinary visits and antimicrobial prescriptions [7]. These conditions are often recurrent and require systemic antimicrobial treatment, frequently initiated empirically, contributing to the selection of resistant bacterial populations [8].

The most common causative agent of canine bacterial skin infections is *Staphylococcus pseudintermedius*, a member of the *Staphylococcus intermedius* group. This Gram-positive, coagulase-positive bacterium is a major component of the normal skin and mucosal microbiota of dogs; however, it can also act as an opportunistic pathogen, causing superficial and deep pyoderma, otitis externa, and various soft tissue infections. The transition from colonization to infection may be facilitated by impairment of the skin barrier, immunological factors, or other predisposing conditions. These include allergic skin diseases (e.g., atopic dermatitis), ectoparasite infestations, endocrine disorders, or mechanical disruption of the skin, which collectively promote bacterial overgrowth and invasion [8,9,10].

A wide range of antimicrobials may be considered as therapeutic options in dermatological infections. β-lactams—including penicillin derivatives and cephalosporins—are commonly used as first-line agents in clinical practice; however, other antimicrobial classes may also be used depending on the clinical indication [7,8,11]. Several of these agents are also classified as important or critically important in human medicine. Their use contributes to selective pressure on the skin microbiota, promoting the emergence and persistence of resistant strains [12,13].

The clinical importance of methicillin-resistant *Staphylococcus pseudintermedius* (MRSP) lies primarily in its phenotypic resistance to β-lactam antibiotics, which is routinely identified using oxacillin susceptibility testing. This resistance substantially limits the therapeutic applicability of β-lactams [8,9]. The molecular basis of this resistance is most commonly associated with the presence of the *mecA* gene, which is located on the staphylococcal cassette chromosome mec (SCCmec) and encodes the altered penicillin-binding protein (PBP2a), thereby reducing the affinity of β-lactam antibiotics [14,15,16]. The *mecC* gene has also been associated with methicillin resistance; however, according to current knowledge, it has not been reported in *S. pseudintermedius* [17].

Laboratory identification of MRSP is routinely based on phenotypic testing using oxacillin, which remains the standard method for the clinical classification of methicillin resistance [18,19]. Oxacillin-resistant isolates are considered clinically resistant to all β-lactam antibiotics [20]. However, oxacillin-susceptible but *mecA*-positive isolates have also been reported, which may be explained by heterogeneous gene expression or regulatory mechanisms [21,22]. Therefore, molecular methods—particularly detection of the *mecA* gene—may be used as complementary tools alongside phenotypic methods, especially for confirming methicillin resistance and resolving discrepant results [19,23].

The clinical significance of MRSP extends beyond β-lactam resistance, as these isolates frequently exhibit multidrug-resistant (MDR) phenotypes involving multiple antimicrobial classes [24,25]. Multidrug resistance, defined as non-susceptibility to at least one agent in three or more antimicrobial classes [26], represents a major clinical concern in both human and veterinary medicine. In addition, extensively drug-resistant (XDR) and pandrug-resistant (PDR) phenotypes have also been reported in staphylococcal infections, further limiting therapeutic options [27,28].

Despite the increasing number of studies on antimicrobial resistance in *S. pseudintermedius*, important gaps remain in the combined evaluation of phenotypic susceptibility patterns and molecular determinants of methicillin resistance. Several studies have investigated either antimicrobial susceptibility profiles or molecular resistance determinants in *S. pseudintermedius*, and genotype–phenotype relationships have been explored in selected contexts [29,30,31]. However, integrated analyses linking MIC-based susceptibility data with targeted detection of *mec* genes in clinical canine isolates remain relatively limited, particularly at the regional level. Such combined phenotypic–genotypic approaches are essential for improving the interpretation of methicillin resistance and for providing more clinically relevant resistance data.

The aim of the present study was to provide a comprehensive characterization of antimicrobial resistance patterns in clinical canine *S. pseudintermedius* isolates, with particular emphasis on methicillin resistance and multidrug resistance. Antimicrobial susceptibility was assessed using MIC determination, methicillin resistance was evaluated phenotypically, and PCR-based detection of *mecA* and *mecC* genes was performed. In addition, the distribution of multidrug resistance phenotypes was evaluated. The study applies a combined phenotypic and genotypic approach to improve the characterization and interpretation of methicillin resistance and to support antimicrobial stewardship in small animal practice.

## 2. Results

### 2.1. Distribution of Isolates

A total of 243 *Staphylococcus pseudintermedius* isolates were included in the study. Based on oxacillin susceptibility, 47 isolates (19.3%) were classified as methicillin-resistant (MRSP), while 196 isolates (80.7%) were considered methicillin-susceptible (MSSP).

### 2.2. Antimicrobial Susceptibility Profiles

The antimicrobial susceptibility patterns of the isolates are summarized in Table 1, while detailed MIC distributions are provided in Supplementary Table S1.

High levels of resistance were observed for several antimicrobial classes, particularly among β-lactams and tetracyclines, where resistance was widespread across the isolate population.

Macrolides and lincosamides also demonstrated considerable resistance, with a substantial proportion of isolates classified as non-susceptible to these agents.

In contrast, aminoglycosides and phenicols generally exhibited good in vitro activity. Amikacin and florfenicol showed particularly favorable susceptibility profiles, while rifampicin and vancomycin remained highly active, with resistance detected only in few isolates.

Fluoroquinolones showed variable efficacy, with notable differences between individual agents.

MIC_50_ and MIC_90_ values were consistent with these findings, with elevated MIC_90_ values observed for several β-lactams and macrolides, whereas lower MIC_90_ values were detected for aminoglycosides and phenicols.

### 2.3. Comparison of MSSP and MRSP Isolates

Differences in antimicrobial susceptibility patterns between MSSP and MRSP isolates are presented in Table 2, while detailed MIC distributions are provided in Supplementary Tables S2 and S3.

In MSSP isolates, β-lactam antibiotics demonstrated high levels of in vitro activity. Amoxicillin–clavulanic acid, cephalexin, and cefovecin showed particularly high susceptibility rates. In MRSP isolates, resistance to β-lactam antibiotics was consistently observed, in line with their classification based on oxacillin resistance. However, a small number of isolates exhibited in vitro susceptibility to certain β-lactam antibiotics; these findings should be interpreted with caution, as methicillin-resistant staphylococci are considered intrinsically resistant to all β-lactam agents due to the presence of *mecA*-mediated PBP2a, which reduces β-lactam binding affinity.

Beyond β-lactam antibiotics, clear differences were observed across multiple antimicrobial classes. Tetracyclines, macrolides, and lincosamides showed high resistance rates among MRSP isolates, whereas MSSP isolates remained more susceptible to these agents. Aminoglycosides showed variable efficacy, with amikacin retaining higher activity compared to gentamicin and tobramycin, particularly among MRSP isolates. Similarly, fluoroquinolones exhibited heterogeneous activity, with pradofloxacin showing comparatively lower resistance rates than enrofloxacin and marbofloxacin. Among the tested antimicrobials, florfenicol maintained high activity in both MSSP and MRSP isolates, with no resistance detected among MRSP isolates. Rifampicin and vancomycin also demonstrated high in vitro activity, although reduced susceptibility was observed in a subset of MRSP isolates.

### 2.4. Detection of mecA and mecC Genes

PCR analysis targeting the *mecA* and *mecC* genes was performed on all phenotypically oxacillin-resistant isolates (n = 47) to confirm the genetic background of methicillin resistance.

The *mecA* gene was detected in 38 of the 47 isolates (80.9%). Among these, 35 isolates were positive with both PCR systems, while one isolate was positive only with the Aklilu primer set [33] and two isolates only with the Stegger primer set [34]. Overall, 36 isolates were *mecA*-positive using the Aklilu primer set and 37 using the Stegger primer set.

Nine isolates (19.1%) were negative for *mecA* with both PCR systems. The *mecC* gene was not detected in any of the examined isolates.

### 2.5. Multidrug Resistance Patterns

**Figure 1:**
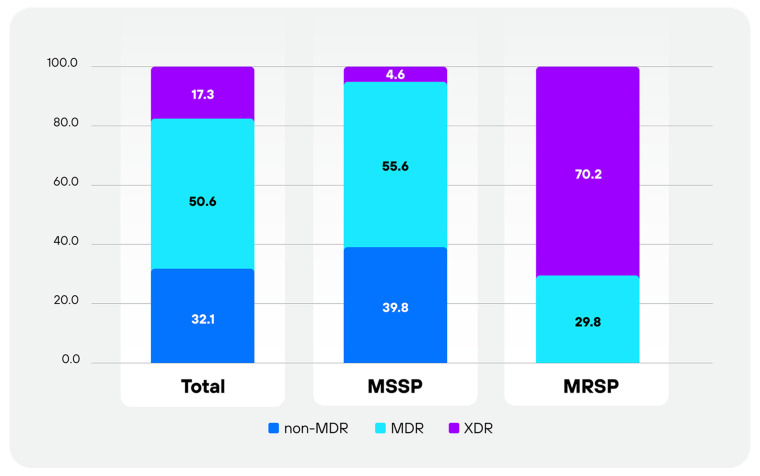
Distribution (%) of antimicrobial resistance categories (non-MDR, MDR and XDR) among total (n = 243), MSSP (n = 196), and MRSP (n = 47) isolates.

The distribution of resistance categories is illustrated in Figure 1 and summarized in Table 3. Detailed counts of isolates according to the number of antimicrobial classes to which they were non-susceptible are provided in Table 4.

A high proportion of isolates exhibited resistance to multiple antimicrobial classes. Based on mutually exclusive categorization, 50.6% of isolates were classified as MDR and 17.3% as XDR, while no PDR isolates were identified. Overall, 67.9% of isolates exhibited resistance to three or more antimicrobial classes.

Clear differences were observed between MSSP and MRSP isolates (Table 3), with statistically significant differences in the distribution of non-MDR, MDR, and XDR categories (*p* < 0.0001). Among MSSP isolates, 55.6% were classified as MDR and 4.6% as XDR, resulting in 60.2% exhibiting resistance to multiple antimicrobial classes. In contrast, all MRSP isolates (100%) were resistant to three or more antimicrobial classes, with a predominance of XDR phenotypes (70.2%).

Analysis of the number of antimicrobial classes to which isolates were non-susceptible revealed a clear shift towards higher resistance categories among MRSP isolates (Table 4). While MSSP isolates were predominantly associated with resistance to fewer antimicrobial classes, MRSP isolates were mainly characterized by resistance to eight or more classes. Notably, MRSP isolates clustered in the highest observed resistance categories (8–9 classes), indicating extensively drug-resistant (XDR) but no pandrug-resistant (PDR) profiles.

Despite the high proportion of XDR isolates, susceptibility to several individual antimicrobial agents was retained. Even among XDR isolates, florfenicol, amikacin, rifampicin, and vancomycin retained high in vitro activity.

### 2.6. Wild-Type and Non-Wild-Type Distribution

The distribution of wild-type (WT) and non-wild-type (NWT) isolates based on EUCAST epidemiological cut-off values is summarized in Table 5.

Overall, a high proportion of isolates were classified as NWT for several antimicrobial agents, indicating the widespread presence of acquired resistance mechanisms within the population. Notably, high NWT proportions were observed for amoxicillin and trimethoprim–sulfamethoxazole.

Marked differences were observed between MSSP and MRSP isolates. MRSP isolates were predominantly classified as NWT across most antimicrobial agents, reflecting a broad distribution of acquired resistance traits. In contrast, MSSP isolates retained higher proportions of WT phenotypes for selected agents, including cephalexin and enrofloxacin.

Florfenicol demonstrated consistently high WT proportions across all groups, and no NWT isolates were detected among MRSP isolates, highlighting its preserved activity.

Overall, ECOFF-based classification provided additional resolution beyond clinical breakpoints, revealing underlying resistance mechanisms not always apparent from S/I/R categorization alone.

## 3. Discussion

### 3.1. Overall Study Context and Analytical Approach

The present study provides a phenotypic and targeted genotypic characterization of antimicrobial resistance in clinical canine *Staphylococcus pseudintermedius* isolates, with particular emphasis on differences between MSSP and MRSP populations. Antimicrobial susceptibility was assessed using MIC-based methods across a broad panel of agents, and PCR-based detection of *mec* genes was performed for phenotypically oxacillin-resistant isolates. The separate evaluation of MSSP and MRSP isolates allows a more detailed interpretation of resistance patterns and their potential clinical relevance.

### 3.2. Prevalence of Methicillin-Resistant Staphylococcus pseudintermedius

The proportion of MRSP isolates detected in the present study (19.3%) is higher than that reported in some previous investigations; however, meaningful comparison between studies requires careful consideration of differences in study design and isolate selection criteria. In Finland, Grönthal et al. reported an MRSP proportion of 13.7% based on clinical diagnostic submissions [23], while a recent German study found a lower prevalence of 7.1% in a large diagnostic dataset [35].

Studies focusing specifically on canine pyoderma may yield higher estimates, as they are enriched for dermatological infections and often include dogs with prior antimicrobial exposure. MRSP has been detected both at infection and colonization sites and may persist after treatment, supporting the notion that dermatological case selection favors the detection of resistant strains [36].

In the present study, isolates were derived from routine diagnostic submissions without predefined inclusion criteria, which were likely enriched for clinically significant or treatment-refractory cases. Consequently, the observed MRSP prevalence and resistance rates may be influenced by this selection bias and may not accurately reflect the situation in the general canine population. Accordingly, the results should be interpreted with caution when extrapolating to broader populations or comparing with systematically sampled datasets, and may represent a higher-end estimate of resistance levels in clinical settings.

### 3.3. Comparative Antimicrobial Resistance Profiles of MSSP and MRSP Isolates

The separate evaluation of MSSP and MRSP isolates revealed marked and clinically relevant differences in antimicrobial susceptibility. MRSP isolates exhibited substantially higher resistance across multiple antimicrobial classes, particularly lincosamides, tetracyclines, fluoroquinolones, and potentiated sulfonamides, indicating a broad multidrug-resistant phenotype.

These findings are consistent with previous studies demonstrating markedly higher resistance levels in MRSP compared to MSSP isolates across diverse clinical datasets [23,35]. Data derived from canine dermatological infections further support this pattern, highlighting extensive co-resistance in MRSP isolates and substantially higher susceptibility in MSSP populations [36,37].

MIC-based investigations also confirm this distinction, showing elevated MIC values and reduced susceptibility in MRSP isolates across multiple antimicrobial classes, reinforcing the close association between methicillin resistance and broader antimicrobial resistance patterns in *S. pseudintermedius* [38].

Despite this general pattern, occasional deviations have been reported. In particular, certain MRSP lineages with lower oxacillin MICs may exhibit in vitro susceptibility to selected β-lactams, indicating that resistance expression is not always uniform [38]. These findings suggest that β-lactam resistance in MRSP may vary depending on strain-specific factors and MIC distributions, occasionally resulting in susceptible or intermediate classifications under in vitro conditions.

In addition, the interpretation of susceptibility results may be influenced by the use of non-veterinary clinical breakpoints for certain antimicrobial agents. Due to the lack of established veterinary-specific criteria, human or surrogate breakpoints were applied in accordance with available CLSI guidelines. While this approach is widely used in veterinary studies, it may affect the classification of isolates as susceptible, intermediate, or resistant. Differences in pharmacokinetic and pharmacodynamic parameters, as well as clinical efficacy, may lead to discrepancies in resistance categorization. Accordingly, resistance data should be interpreted with caution, particularly when comparing studies using different breakpoint systems or when extrapolating findings to clinical decision-making in veterinary practice.

### 3.4. Distribution of Multidrug Resistance Phenotypes in MSSP and MRSP

The close association between methicillin resistance and broader antimicrobial non-susceptibility was also reflected in the overall distribution of resistance phenotypes. More than two-thirds of isolates exhibited resistance to at least three or more antimicrobial classes, indicating a high overall burden of multidrug resistance.

When analyzed according to methicillin phenotype, clear and statistically significant differences became apparent. Among MSSP isolates, the majority (55.6%) exhibited MDR phenotypes, whereas XDR phenotypes were relatively uncommon (4.6%). In contrast, all MRSP isolates were resistant to multiple antimicrobial classes, with a predominance of XDR phenotypes (70.2%), highlighting the strong link between methicillin resistance and extensively drug-resistant phenotypes.

Direct comparison with the literature requires caution, as most studies report multidrug resistance without further stratification. Nevertheless, the overall burden of multidrug resistance observed in the present study is consistent with previous reports demonstrating extensive resistance in MRSP isolates, particularly in canine dermatological infections [37,39]. The absence of PDR isolates, together with the high proportion of XDR phenotypes, suggests that although resistance levels are extensive, complete resistance to all antimicrobial classes remains uncommon. This pattern indicates that, despite advanced resistance, some therapeutic options may still be preserved. Detailed stratification into advanced resistance categories (XDR, PDR) remains limited in the veterinary literature; therefore, the present findings provide additional resolution on the spectrum of resistance phenotypes in *S. pseudintermedius* populations.

It should be noted that the classification of multidrug resistance in the present study was performed in accordance with the widely accepted criteria proposed by Magiorakos et al. [26], in which antimicrobial agents are grouped at the class level. Accordingly, β-lactam antibiotics were considered as a single antimicrobial class, reflecting their shared mechanism of action and common resistance determinant.

This classification framework is intended to assess the overall burden of acquired resistance rather than the clinical efficacy of individual agents. Consequently, non-susceptibility to a single agent results in classification of the entire class as resistant, even if susceptibility to other agents within that class is retained. Thus, MDR and XDR categories represent standardized epidemiological descriptors rather than direct indicators of therapeutic options.

### 3.5. Molecular Basis of Methicillin Resistance: mecA and mecC Findings

In *S. pseudintermedius*, methicillin resistance is primarily mediated by the *mecA* gene, which encodes the low-affinity penicillin-binding protein PBP2a and is carried on the staphylococcal cassette chromosome mec (SCCmec) [14]. SCCmec is a structurally diverse mobile genetic element containing the mec gene complex and cassette chromosome recombinase (*ccr*) genes, often with additional resistance-associated sequences [40,41].

In the present study, the *mecA* detection rate was lower than the number of phenotypically oxacillin-resistant isolates, consistent with previously described genotype–phenotype discordance in canine *S. pseudintermedius*. Oxacillin-susceptible *mecA*-positive isolates have been reported, while *mecA* may also be absent in phenotypically resistant isolates, indicating incomplete concordance between phenotypic and molecular methods [22,42]. This discordance may result from heterogeneous *mecA* expression, sequence variation within the *mec* gene complex, and structural diversity of SCCmec elements affecting both phenotypic expression and molecular detection [14,21,22,41,43]. Primer specificity in PCR assays may further limit detection of *mecA* variants, while the use of two PCR systems in this study likely improved detection sensitivity.

No *mecC*-positive isolates were identified, supporting evidence that methicillin resistance in *S. pseudintermedius* is predominantly *mecA*-mediated [44].

PCR-based detection confirms the primary genetic determinant of methicillin resistance but provides limited information on SCCmec structure or strain background. As molecular analysis was restricted to phenotypically methicillin-resistant isolates, oxacillin-susceptible *mecA*-positive strains may have been missed, and alternative resistance mechanisms were not investigated. Furthermore, the analysis was limited to *mecA* and *mecC* detection without characterization of SCCmec types, clonal lineages, or additional resistance determinants. While sufficient for confirming the primary mechanism, this approach provides limited insight into the epidemiological and genetic context of multidrug resistance. More comprehensive approaches, such as SCCmec typing, multilocus sequence typing (MLST), or whole-genome sequencing, would enable deeper characterization.

### 3.6. Antimicrobial Susceptibility Patterns in MSSP Isolates

Beyond the general comparison between MSSP and MRSP isolates, separate evaluation of the MSSP population provides insight into antimicrobial agents that may remain clinically useful in methicillin-susceptible infections.

Among β-lactam antibiotics, amoxicillin–clavulanic acid, cephalexin and cefovecin showed consistently high activity against MSSP isolates, with susceptibility rates exceeding 90%. Similar findings have been reported in previous studies, including canine superficial bacterial pyoderma, where susceptibility was highest for cephalexin and amoxicillin–clavulanic acid (both 96%) [45], and in large diagnostic datasets confirming preserved activity of first-generation cephalosporins [35]. These data support the continued role of β-lactamase-stable β-lactams as first-line therapeutic options in MSSP infections.

In contrast, β-lactams lacking β-lactamase inhibitors showed markedly reduced efficacy, with resistance rates exceeding 50%, consistent with reports of high aminopenicillin resistance among canine isolates [35]. These findings indicate that non-protected β-lactams are unreliable even in methicillin-susceptible populations.

Among non-β-lactam agents, a heterogeneous susceptibility pattern was observed. Clindamycin, tetracyclines and trimethoprim–sulfamethoxazole showed moderate and variable activity, with resistance levels ranging widely across studies, including lower non-susceptibility in Northern European datasets [23], variability associated with prior antimicrobial exposure [46], reduced susceptibility in some regional populations [47], and evidence of increasing resistance trends over time [48]. These findings indicate that the clinical performance of these agents may be less predictable than that of β-lactams and may depend strongly on local resistance patterns.

Fluoroquinolone susceptibility patterns in the present study are of particular concern. While pradofloxacin retained relatively favorable activity, enrofloxacin and marbofloxacin showed reduced efficacy and considerable proportions of non-susceptible isolates. In comparison, Finnish MSSP isolates showed only 2.6% non-susceptibility to enrofloxacin [23], and no resistance was detected in isolates from healthy dogs in Canada [49]. However, higher resistance levels have been reported in other settings, including studies where resistance approached or exceeded 30–40% [50]. These findings may reflect a shift in susceptibility patterns within MSSP populations. Given that fluoroquinolones are classified as AMEG B Category (“Restrict”) antimicrobials [51], these observations have direct implications for antimicrobial stewardship and warrant cautious clinical use.

Within the aminoglycoside and phenicol classes, amikacin and florfenicol retained high activity, whereas other agents (e.g., gentamicin, tobramycin, chloramphenicol) showed more variable susceptibility. Similar patterns have been reported previously, including preserved amikacin activity against resistant strains [23] and variable phenicol susceptibility across populations [48,49]. These findings indicate that antimicrobial activity cannot be generalized at the class level and should be interpreted on an agent-specific basis.

Taken together, these observations indicate that MSSP isolates retain susceptibility to several antimicrobial agents; however, marked differences between antimicrobial classes and increasing variability across studies underscore the importance of culture and susceptibility testing to guide therapy, even in methicillin-susceptible infections.

### 3.7. Comparison of ECOFF-Based and Clinical Susceptibility Classifications

In addition to clinical breakpoint-based interpretation, isolates were also evaluated using epidemiological cut-off values (ECOFFs), where available, to assess susceptibility patterns at the population level. Differences between WT/NWT and S/I/R classifications were observed for several antimicrobial agents. In particular, certain agents with relatively favorable susceptibility rates still showed a high proportion of NWT isolates. For example, trimethoprim–sulfamethoxazole demonstrated 81.1% susceptibility, yet 77.8% of isolates were classified as non-wild-type.

Similarly, for fluoroquinolones, a substantial proportion of isolates classified as susceptible or intermediate were categorized as NWT, reflecting elevated MIC values within the population. These discrepancies indicate that clinical susceptibility may coexist with underlying acquired resistance mechanisms that are not yet reflected in clinical categorization.

These findings suggest that ECOFF-based classification can detect early shifts in susceptibility distributions not yet captured by clinical breakpoints. This is particularly relevant for antimicrobial classes showing reduced or intermediate susceptibility, indicating a gradual population-level shift towards decreased susceptibility. Accordingly, ECOFF-based interpretation may support surveillance and antimicrobial stewardship by enabling earlier recognition of emerging resistance trends.

Importantly, ECOFF-based and clinical breakpoint-based classifications should be interpreted in a complementary manner rather than interchangeably. ECOFFs are particularly useful for detecting the emergence and spread of acquired resistance mechanisms at the population level, whereas clinical breakpoints remain essential for guiding therapeutic decision-making. Consequently, isolates classified as non-wild-type may still be considered clinically susceptible, and this distinction should be considered when translating susceptibility data into clinical practice.

### 3.8. Antimicrobial Agents with Preserved Activity

Taken together, the susceptibility patterns observed in the present study indicate that certain antimicrobial agents retained notable activity and may remain clinically relevant in selected cases despite the substantial burden of multidrug resistance.

Florfenicol demonstrated particularly favorable activity, with a very low overall resistance rate (2.4%) and no resistant MRSP isolates identified. Similar observations have been reported previously, including in multidrug-resistant *S. pseudintermedius* populations [23]. In contrast, chloramphenicol resistance was substantially higher, reflecting differences in resistance mechanisms. Chloramphenicol resistance is typically mediated by enzymatic inactivation, whereas florfenicol is structurally protected from this mechanism. Additional resistance determinants, such as *cfr*-mediated ribosomal methylation or efflux systems (e.g., *floR*), may contribute to phenicol resistance but appear to be less prevalent in companion animal isolates [52,53]. The absence of florfenicol resistance among MRSP isolates may reflect the limited association of phenicol resistance determinants with SCCmec-linked multidrug resistance platforms [25,52]. MIC-based observations further support this pattern, indicating that the presence of resistance genes does not always translate into phenotypic resistance, as demonstrated in isolates carrying phenicol resistance determinants without elevated florfenicol MIC values [54]. In addition, the limited use of florfenicol in companion animals may reduce selective pressure, contributing to the low prevalence of resistance [52].

Aminoglycosides demonstrated variable activity; however, amikacin consistently showed the highest efficacy across all groups, including MRSP isolates. This observation is consistent with previous reports demonstrating lower resistance rates for amikacin compared to other aminoglycosides [23]. The favorable activity of amikacin can be attributed to its structural resistance to most aminoglycoside-modifying enzymes, which commonly mediate resistance within this class [55,56]. Although its clinical use may be limited by parenteral administration and potential toxicity, its retained activity suggests a potential role as a reserve option in selected multidrug-resistant infections.

Rifampicin and vancomycin are antimicrobials of critical importance in human medicine, and their use in veterinary practice is highly restricted. In the European Union, glycopeptides, including vancomycin, are reserved exclusively for human use under Commission Implementing Regulation (EU) 2022/125 [57]. Rifampicin is classified as an AMEG A Category (“Avoid”) antimicrobial, and its use is limited to exceptional circumstances via the drug cascade [51]. In the present study, both agents demonstrated high in vitro activity, with very low resistance rates. These findings are consistent with previous reports indicating preserved susceptibility in *S. pseudintermedius* [58]. However, this apparent efficacy should be interpreted strictly within an epidemiological context and does not translate into routine clinical applicability in veterinary practice. Rifampicin resistance is primarily associated with mutations in the *rpoB* gene and may emerge rapidly under selective pressure [59]. Vancomycin resistance has not been clearly established in *S. pseudintermedius*, and susceptibility appears to be largely preserved, likely reflecting the absence of selective pressure in veterinary settings [60].

## 4. Materials and Methods

### 4.1. Bacterial Isolates

A total of 243 *Staphylococcus pseudintermedius* isolates were included in this study. The isolates were obtained from the DUO-BAKT Veterinary Microbiological Laboratory (Veresegyház, Hungary). All isolates were clinical isolates recovered from canine skin samples.

The samples were originally collected by practicing veterinarians during routine clinical examinations and submitted to the diagnostic laboratory for microbiological analysis. The isolates included in this study were identified between 2023 and 2025 during routine diagnostic procedures; however, only a subset of the isolates processed by the laboratory during this period was made available for the present study. The isolates analyzed in this study were selected based on availability and were provided by the diagnostic laboratory without predefined inclusion criteria.

Bacterial identification was performed using matrix-assisted laser desorption/ionization time-of-flight mass spectrometry (MALDI-TOF MS) (Bruker Daltonik GmbH, Bremen, Germany).

Following identification, the isolates were stored in a mixture of 800 µL of tryptone soy broth (PharmaBio Tryptone Soy Broth, Biolab Zrt., Budapest, Hungary) and 200 µL of sterile glycerol (Glycerol 87% P.A., Lach-Ner, Ltd., Neratovice, Czech Republic) at −80 °C, at the Microbiology Laboratory of the Department of Pharmacology and Toxicology, University of Veterinary Medicine Budapest.

The results of the present study should be interpreted with caution when extrapolating to the national level, as the analyzed isolates do not constitute a representative sample of the overall canine population.

### 4.2. Antimicrobial Susceptibility Testing

The antimicrobial susceptibility of the isolates was assessed by determining minimum inhibitory concentration (MIC) values using the broth microdilution method, in accordance with the guidelines of the Clinical and Laboratory Standards Institute (CLSI VET01, 6th edition, 2024) [61].

The antimicrobial panel comprised penicillin (PEN), oxacillin (OXA), amoxicillin (AMX), amoxicillin–clavulanic acid (AMC, 2:1 ratio), cephalexin (CEFX), ceftazidime (CAZ), cefovecin (COV), imipenem (IPM), gentamicin (GEN), tobramycin (TOB), amikacin (AMK), oxytetracycline (OTC), doxycycline (DOX), azithromycin (AZM), rifampicin (RIF), clindamycin (CLI), florfenicol (FFC), chloramphenicol (CHL), ciprofloxacin (CIP), enrofloxacin (ENR), marbofloxacin (MAR), pradofloxacin (PRA), trimethoprim–sulfamethoxazole (SXT, 1:19 ratio), and vancomycin (VAN).

All antimicrobial agents were obtained from Sigma-Aldrich (St. Louis, MO, USA), with the exception of cefovecin, which was purchased from LGC (Teddington, UK), and pradofloxacin, which was obtained from Biosynth (Staad, Switzerland).

Stock solutions of the antimicrobial agents were prepared and subsequently diluted in cation-adjusted Mueller–Hinton broth (CAMHB; Biolab Ltd., Budapest, Hungary). Two-fold serial dilutions were performed in 96-well microplates (BRANDplates, pureGrade S; VWR International, Radnor, PA, USA) to achieve the required concentration ranges.

Bacterial inocula were prepared in CAMHB and adjusted to yield a final concentration of approximately 5 × 10^5^ colony-forming units (CFU)/mL in each well. The microplates were incubated at 35 °C under aerobic conditions in accordance with CLSI (CLSI VET01S, 7th edition, 2024) [32] recommendations, as incubation at higher temperatures may impair the detection of methicillin-resistant staphylococci. The incubation time was 16–20 h for most antimicrobial agents, while oxacillin and vancomycin plates were incubated for 24 h.

MIC values were defined as the lowest concentrations of antimicrobial agents that completely inhibited visible bacterial growth.

Quality control was performed using the reference strain *Staphylococcus aureus* ATCC 29213 (American Type Culture Collection, Manassas, VA, USA), in accordance with CLSI VET01S (7th edition, 2024) guidelines.

### 4.3. Interpretation of MIC Values

Interpretation of MIC values into susceptible (S), intermediate (I), and resistant (R) categories was performed in accordance with the Clinical and Laboratory Standards Institute guidelines (CLSI VET01S, 7th edition, 2024) [32]. Where available, canine-specific clinical breakpoints were applied. In cases where veterinary breakpoints were not available, human clinical breakpoints were used, specifically for oxacillin, gentamicin, rifampicin, trimethoprim–sulfamethoxazole, and vancomycin. For penicillin, equine-specific clinical breakpoints were applied. The breakpoints applied for each antimicrobial agent are summarized in Supplementary Table S4.

For certain antimicrobial agents, surrogate breakpoints were applied due to the lack of specific veterinary interpretive criteria. The canine clinical breakpoint established for gentamicin was used for tobramycin, while the breakpoint established for chloramphenicol was applied to florfenicol.

For ceftazidime, imipenem, and ciprofloxacin, no CLSI clinical breakpoints were available; therefore, isolates tested against these agents were not interpreted using S/I/R categories.

In addition to clinical categorization, isolates were classified as wild-type (WT) or non-wild-type (NWT) based on epidemiological cut-off values (ECOFFs) obtained from the EUCAST database [62]. This classification was used to assess the presence of acquired resistance mechanisms independently of clinical breakpoints. The ECOFF values applied for each antimicrobial agent are summarized in Supplementary Table S5.

### 4.4. Methicillin Resistance Classification

Methicillin resistance was defined based on oxacillin MIC values determined by broth microdilution. Isolates with an oxacillin MIC ≥ 0.5 µg/mL were classified as methicillin-resistant *Staphylococcus pseudintermedius* (MRSP), while isolates with lower MIC values were considered methicillin-susceptible (MSSP), in accordance with CLSI guidelines (CLSI VET01S, 7th edition, 2024) [32].

All oxacillin-resistant isolates were subsequently subjected to molecular analysis for the detection of the *mecA* and *mecC* genes.

### 4.5. Molecular Detection of mecA and mecC Genes

Nucleic acid was extracted from bacterial cultures grown in cation-adjusted Mueller–Hinton broth (CAMHB) using the Zymo Quick-RNA Fungal/Bacterial Miniprep Kit (Zymo Research, Irvine, CA, USA) according to the manufacturer’s instructions. Briefly, 200 µL of bacterial culture was used for extraction, and the nucleic acid was eluted in 60 µL of elution buffer and stored at −20 °C until further analysis.

Polymerase chain reaction (PCR) assays targeting the *mecA* and *mecC* genes were performed as previously described by Aklilu et al. [33] and Stegger et al. [34]. Separate PCRs were carried out for the detection of *mecA* and *mecC* genes. The primer sequences are listed in Supplementary Table S6. Each PCR was performed in a total volume of 25 µL, containing 1× DreamTaq Green buffer, 200 µM dNTP mix, 200 nM of each primer, 0.75 U DreamTaq DNA Polymerase (Thermo Fisher Scientific, Waltham, MA, USA), and 2 µL of extracted nucleic acid. The thermal cycling conditions were as follows: initial denaturation at 95 °C for 3 min, followed by 45 cycles of denaturation at 95 °C for 30 s, annealing for 30 s (annealing temperatures as in Supplementary Table S6), and extension at 72 °C for 1 min, with a final extension step at 72 °C for 10 min. PCR products were analyzed by agarose gel electrophoresis using 1% agarose gel. PCR analysis was performed on all oxacillin-resistant isolates. Selected PCR amplicons were subjected to Sanger sequencing, and the obtained sequences were confirmed to match reference mecA sequences, thereby providing validation of the specificity of the PCR assay.

### 4.6. Multidrug Resistance Analysis

Multidrug resistance (MDR), extensively drug-resistant (XDR), and pandrug-resistant (PDR) phenotypes were defined according to the criteria proposed by Magiorakos et al. [26].

Antimicrobial agents were grouped into classes based on their mechanism of action in accordance with the principles of Magiorakos et al. [26] (see Supplementary Table S7). β-lactam antibiotics were considered as a single antimicrobial class due to their shared mechanism of action. All tested antimicrobial classes were included in the MDR/XDR classification framework.

Interpretation of resistance for MDR analysis was based on antimicrobial susceptibility testing results. Isolates classified as either intermediate (I) or resistant (R) according to CLSI breakpoints were considered non-susceptible.

The number of antimicrobial classes to which each isolate was non-susceptible was determined, and isolates were categorized as follows: non-MDR (resistant to ≤2 antimicrobial classes), MDR (resistant to ≥3 classes), XDR (non-susceptible to all but ≤2 classes), and PDR (non-susceptible to all tested antimicrobial classes).

Antimicrobial agents for which no clinical breakpoints were available (ceftazidime, imipenem, and ciprofloxacin) were excluded from the MDR analysis.

### 4.7. Data Analysis

MIC values were analyzed descriptively. For each antimicrobial agent, MIC_50_ and MIC_90_ values were calculated for the overall population as well as for MSSP and MRSP isolates separately.

Isolates were categorized as susceptible (S), intermediate (I), or resistant (R) according to CLSI clinical breakpoints [32]. The proportions of isolates in each category were determined for all antimicrobial agents.

Wild-type (WT) and non-wild-type (NWT) populations were defined based on epidemiological cut-off values (ECOFFs) obtained from the EUCAST database [62]. The distribution of WT and NWT isolates was determined for each antimicrobial agent.

Multidrug resistance (MDR), extensively drug-resistant (XDR), and pandrug-resistant (PDR) phenotypes were determined as described above. The number of antimicrobial classes to which each isolate was non-susceptible was calculated, and the distribution of resistance across antimicrobial classes was analyzed for the total population as well as for MSSP and MRSP isolates.

All analyses were performed using Microsoft Excel (Microsoft Corporation, Redmond, WA, USA).

Statistical comparisons between MSSP and MRSP isolates across antimicrobial resistance categories (non-MDR, MDR, XDR, PDR) were performed using Fisher’s exact test. A *p*-value of <0.05 was considered statistically significant.

## 5. Conclusions

The present study provides a detailed phenotypic and targeted genotypic characterization of antimicrobial resistance in clinical canine *S. pseudintermedius* isolates, with particular emphasis on methicillin resistance and multidrug resistance patterns. The results demonstrate that MRSP isolates are associated with markedly broader resistance profiles than MSSP isolates, including substantially higher frequencies of MDR and XDR phenotypes, while no PDR isolates were identified, confirming the major clinical relevance of methicillin resistance in this species.

At the same time, separate evaluation of MSSP isolates showed that susceptibility to several antimicrobial agents remains preserved, particularly for β-lactamase-stable β-lactams, whereas other classes, such as tetracyclines, lincosamides, and fluoroquinolones, displayed more variable activity. The relatively high proportion of resistant and intermediate MSSP isolates observed for some fluoroquinolones is of particular concern, especially in light of their importance in antimicrobial stewardship frameworks. When MDR and XDR phenotypes were considered together, a substantial proportion of isolates (67.9%) exhibited resistance to three or more antimicrobial classes, further highlighting the clinical relevance of multidrug resistance in this population.

Importantly, only a limited number of antimicrobial agents retained activity across both MSSP and MRSP populations, most notably amikacin, florfenicol and rifampicin, highlighting their potential relevance in the management of infections involving multidrug-resistant *S. pseudintermedius*. Vancomycin also demonstrated preserved activity in the majority of isolates; however, its clinical use in veterinary medicine is restricted.

The molecular findings further support the central role of *mecA* in methicillin resistance in canine *S. pseudintermedius*, while also indicating that genotype–phenotype concordance is not always complete. The results also demonstrate that the use of multiple PCR approaches targeting different regions of *mecA* may improve detection in genetically diverse populations.

Overall, these findings reinforce the importance of culture-based antimicrobial susceptibility testing, careful interpretation of methicillin resistance, and continued surveillance of resistance trends in canine *S. pseudintermedius*. Together, the results support more informed therapeutic decision-making and emphasize the relevance of antimicrobial stewardship in the management of canine staphylococcal infections.

## Figures and Tables

**Table 1:** Antimicrobial susceptibility profiles of *Staphylococcus pseudintermedius* isolates (n = 243), including S/I/R distribution and MIC_50_/MIC_90_ values.

Antibiotic	S (%)	I (%)	R (%)	MIC_50_ (µg/mL)	MIC_90_ (µg/mL)
PEN	31.3	5.3	63.4	8	128
OXA	80.2	-	19.8	0.06	64
AMX	32.5	-	67.5	1	64
AMC	74.1	12.3	13.6	0.125	2
CEFX	77.0	-	23.0	1	128
CAZ	-	-	-	2	256
COV	75.7	4.5	19.8	0.125	512
IPM	-	-	-	0.015	0.5
GEN	63.4	2.9	33.7	0.25	32
TOB	65.5	8.6	25.9	0.25	16
AMK	88.9	2.9	8.2	1	8
OTC	43.6	2.5	53.9	16	128
DOX	44.5	1.6	53.9	1	8
AZM	51.0	0.0	49.0	1	512
RIF	95.5	3.3	1.2	<0.015	0.5
CLI	52.6	2.5	44.9	0.125	>512
FFC	94.7	2.9	2.4	1	2
CHL	40.7	12.3	47	4	32
CIP	-	-	-	0.125	32
ENR	49.8	18.5	31.7	0.125	16
MAR	38.3	21.4	40.3	0.25	16
PRA	70.8	15.6	13.6	0.03	2
SXT	65.4	-	34.6	0.5	64
VAN	98.4	0.0	1.6	0.5	1

-: not interpreted due to lack of clinical breakpoints. Abbreviations: PEN, penicillin; OXA, oxacillin; AMX, amoxicillin; AMC, amoxicillin–clavulanic acid; CEFX, cephalexin; CAZ, ceftazidime; COV, cefovecin; IPM, imipenem; GEN, gentamicin; TOB, tobramycin; AMK, amikacin; OTC, oxytetracycline; DOX, doxycycline; AZM, azithromycin; RIF, rifampicin; CLI, clindamycin; FFC, florfenicol; CHL, chloramphenicol; CIP, ciprofloxacin; ENR, enrofloxacin; MAR, marbofloxacin; PRA, pradofloxacin; SXT, trimethoprim–sulfamethoxazole; VAN, vancomycin. Breakpoints were interpreted according to CLSI VET01S (7th ed., 2024) [32]. Where canine-specific breakpoints were not available, human or equine clinical breakpoints were applied. Surrogate criteria were used where indicated. Detailed information on the source of breakpoints (canine, human, equine, or surrogate) is provided in Supplementary Table S4.

**Table 2:** Antimicrobial susceptibility profiles of MSSP (n = 196) and MRSP (n = 47) isolates, including S/I/R distribution and MIC_50_/MIC_90_ values.

Antibiotic	MSSP (n = 196)					MRSP (n = 47)				
	S (%)	I (%)	R (%)	MIC_50_ µg/mL	MIC_90_ µg/mL	S (%)	I (%)	R (%)	MIC_50_ µg/mL	MIC_90_ µg/mL
PEN	38.8	6.6	54.6	4	64	0.0	0.0	100.0	64	256
OXA	100.0	-	0.0	0.06	0.125	0.0	-	100.0	64	512
AMX	40.3	-	59.7	0.5	8	0.0	-	100.0	64	128
AMC	91.8	7.2	1.0	0.125	0.25	0.0	14.9	85.1	2	16
CEFX	95.4	-	4.6	1	2	0.0	-	100.0	128	256
CAZ	-	-	-	2	8	-	-	-	32	512
COV	93.9	2.5	3.6	0.125	0.5	10.7	2.1	87.2	512	>512
IPM	-	-	-	0.015	0.03	-	-	-	0.25	32
GEN	78.6	3.6	17.8	0.125	16	17.0	23.4	59.6	16	32
TOB	81.1	9.2	9.7	0.125	8	27.7	19.1	53.2	16	32
AMK	98	0.5	1.5	1	2	59.6	10.6	29.8	4	128
OTC	54.1	3.1	42.8	0.25	64	12.8	4.3	82.9	64	128
DOX	55.1	2.0	42.9	0.015	8	17.0	0.0	83.0	2	16
AZM	63.3	0.0	36.7	0.25	512	8.5	0.0	91.5	256	512
RIF	98.5	1.0	0.5	<0.015	<0.015	89.4	8.5	2.1	<0.015	1
CLI	65.3	3.1	31.6	0.06	512	6.4	0.0	93.6	512	>512
FFC	97.5	1.0	1.5	1	2	93.6	6.4	0.0	1	2
CHL	50.5	15.3	34.2	2	32	29.8	36.2	34	4	32
CIP	-	-	-	0.06	1	-	-	-	16	64
ENR	61.7	23	15.3	0.06	1	6.4	12.8	80.8	8	256
MAR	47.5	26.5	26	0.25	2	6.4	8.5	85.1	8	32
PRA	87.7	8.7	3.6	0.03	0.5	19.2	40.4	40.4	1	8
SXT	81.1	-	18.9	0.5	16	21.3	-	78.7	16	256
VAN	100.0	0.0	0.0	0.5	1	91.5	0.0	8.5	0.5	2

-: not interpreted due to lack of clinical breakpoints. Abbreviations: PEN, penicillin; OXA, oxacillin; AMX, amoxicillin; AMC, amoxicillin–clavulanic acid; CEFX, cephalexin; CAZ, ceftazidime; COV, cefovecin; IPM, imipenem; GEN, gentamicin; TOB, tobramycin; AMK, amikacin; OTC, oxytetracycline; DOX, doxycycline; AZM, azithromycin; RIF, rifampicin; CLI, clindamycin; FFC, florfenicol; CHL, chloramphenicol; CIP, ciprofloxacin; ENR, enrofloxacin; MAR, marbofloxacin; PRA, pradofloxacin; SXT, trimethoprim–sulfamethoxazole; VAN, vancomycin. Breakpoints were interpreted according to CLSI VET01S (7th ed., 2024) [32]. Where canine-specific breakpoints were not available, human or equine clinical breakpoints were applied. Surrogate criteria were used where indicated. Detailed information on the source of breakpoints (canine, human, equine, or surrogate) is provided in Supplementary Table S4.

**Table 3:** Distribution of isolates across antimicrobial resistance categories based on the number of non-susceptible antimicrobial classes (Magiorakos et al. [26]) in total, MSSP and MRSP isolates, including *p*-values.

Resistance Category	Definition	Total (n = 243)	MSSP (n = 196)	MRSP (n = 47)	*p*-Value
non-MDR	≤2 classes	78 (32.1%)	78 (39.8%)	0 (0%)	<0.0001
MDR	≥3 classes	123 (50.6%)	109 (55.6%)	14 (29.8%)	<0.0001
XDR	susceptible to ≤2 classes	42 (17.3%)	9 (4.6%)	33 (70.2%)	<0.0001
PDR	resistant to all classes	0 (0%)	0 (0%)	0 (0%)	NA

NA: not applicable due to absence of PDR isolates in both groups.

**Table 4:** Distribution of isolates according to the number of antimicrobial classes to which they were non-susceptible.

Number of Classes	Total (n = 243)	MSSP (n = 196)	MRSP (n = 47)
0	13	13	0
1	28	28	0
2	37	37	0
3	28	27	1
4	23	23	0
5	27	24	3
6	22	20	2
7	23	15	8
8	35	8	27
9	7	1	6
10	0	0	0

**Table 5:** Distribution (%) of wild-type (WT) and non-wild-type (NWT) isolates based on epidemiological cut-off values (ECOFFs) among total (n = 243), MSSP (n = 196), and MRSP (n = 47) populations.

Antibiotic	WT% (Total)	NWT% (Total)	WT% (MSSP)	NWT% (MSSP)	WT% (MRSP)	NWT% (MRSP)
AMX	11.9	88.1	14.8	85.2	0.0	100.0
CEFX	77.4	22.6	95.9	4.1	8.5	91.5
GEN	57.6	42.4	71.4	28.6	6.4	93.6
OTC	46.1	53.9	57.1	42.9	17.0	83.0
DOX	44.4	55.6	55.1	44.9	17.0	83.0
CLI	52.3	47.7	64.8	35.2	4.3	95.7
FFC	97.5	2.5	98.5	1.5	100.0	0.0
ENR	70.4	29.6	87.2	12.8	25.5	74.5
SXT	22.2	77.8	27.6	72.4	2.1	97.9

## Data Availability

The original contributions presented in this study are included in the article/Supplementary Materials. Further inquiries can be directed to the corresponding author.

## Supplementary Materials

The following supporting information can be downloaded at https://www.mdpi.com/article/10.3390/antibiotics15050473/s1, Table S1: MIC distribution of antimicrobial agents against *Staphylococcus pseudintermedius* isolates (n = 243), including MIC_50_, MIC_90_ values; red lines indicate clinical resistance breakpoints (CLSI VET01S, 7th edition, 2024 [32]). Table S2: MIC distribution of antimicrobial agents against methicillin-susceptible *Staphylococcus pseudintermedius* (MSSP) isolates (n = 196), including MIC_50_, MIC_90_ values; red lines indicate clinical resistance breakpoints (CLSI VET01S, 7th edition, 2024 [32]). Table S3: MIC distribution of antimicrobial agents against methicillin-resistant *Staphylococcus pseudintermedius* (MRSP) isolates (n = 47), including MIC_50_, MIC_90_ values; red lines indicate clinical resistance breakpoints (CLSI VET01S, 7th edition, 2024 [32]). Table S4: Clinical breakpoints used for antimicrobial susceptibility interpretation of canine *Staphylococcus pseudintermedius* isolates according to CLSI VET01S (7th edition, 2024 [32]). Table S5: Epidemiological cut-off values (ECOFFs) for *Staphylococcus pseudintermedius* provided by EUCAST, used for wild-type and non-wild-type classification. Table S6: Oligonucleotides used for mecA and mecC gene specific polymerase chain reaction in this study. Table S7: Classification of antimicrobial agents into classes used for multidrug resistance (MDR) analysis.
