# Supplementary material for: Comparative Phenotypic and PCR-Based Methicillin Resistance Characterization of Clinical Canine Staphylococcus pseudintermedius
Source: Antibiotics (Basel). 2026 May 7;15(5):473. doi: 10.3390/antibiotics15050473 (PMC13203898; doi:10.3390/antibiotics15050473)
Supplement: Supplementary file 1 [file antibiotics-15-00473-s001.zip › antibiotics-4291756-supplementary.pdf]

Supplementary Table S1. MIC distribution of antimicrobial agents against *Staphylococcus pseudintermedius* isolates (n = 243), including MIC_{50}, MIC_{90} values; red lines indicate clinical resistance breakpoints (CLSI VET01S, 7th edition, 2024).

Antibiotics	≥128	64	32	16	8	4	2	1	0.5	0.25	0.125	0.06	0.03	≤0.015	MIC_{50} (µg/mL)	MIC_{90} (µg/mL)
PEN[e]	49	39	11	16	18	13	8	13	5	13	9	7	7	35	8	128
OXA*	22	3	8	5			2	2	6	11	35	111	34	4	0.06	64
AMX	8	20	12	25	18	15	21	25	20	27	10	13	14	15	1	64
AMC	4		1	5	8	6	4	5	30	41	71	34	22	12	0.125	2
CEFX	32	8	2	6	7	2	22	99	63			2			1	128
CAZ	31	8	16	9	8	8	86	75	2						2	256
COV	36			3	4	3	2	11	9	33	88	50	3	1	0.125	512
IPM	2	1	3	1	4	1	2	6	11	8	6	13	22	163	0.015	0.5
GEN*	3	3	29	47	7	3		1	10	27	78	29	1	5	0.25	32
TOB+	3	2	10	48	21	8	1		4	31	71	39		5	0.25	16
AMK	12	6		2	7	37	48	111	17	2		1			1	8
OTC	37	44	33	12	3	1		1	6	19	53	25	6	3	16	128
DOX		3	1	8	59	21	21	12	6	4	1	2	3	102	1	8
AZM*	112	7					1	2	10	54	36	17	3	1	1	512
RIF*						3	8	5	10	2			1	214	<0.015	0.5
CLI	95	6	3	3	2		3	3	1	1	12	44	54	16	0.125	>512
FFC	2			4		7	82	141	7						1	2
CHL+		6	95	12	1	30	93	6							4	32
CIP	8	9	13	15	14	3	3	3	13	13	48	61	37	3	0.125	32
ENR	10	4	10	6	18	15	1	8	5	10	35	68	44	9	0.125	16
MAR	4		13	36	11	3	2	9	20	52	72	14	2	5	0.25	16
PRA				1	7	2	23	32	6	5	3	36	105	23	0.03	2
SXT*	20	15	19	17	8	5	9	26	34	36	39	14	1		0.5	64
VAN*	3		1				12	61	87	71	7	1			0.5	1

Breakpoints were interpreted according to CLSI VET01S (7th edition, 2024). *Based on human clinical breakpoint. [e]Based on equine clinical breakpoint. +The canine clinical breakpoint established for gentamicin was applied to tobramycin, while the canine clinical breakpoint established for chloramphenicol was applied to florfenicol.

Supplementary Table S2. MIC distribution of antimicrobial agents against methicillin-susceptible *Staphylococcus pseudintermedius* (MSSP) isolates (n = 196), including MIC_{50}, MIC_{90} values; red lines indicate clinical resistance breakpoints (CLSI VET01S, 7th edition, 2024).

Antibiotics	≥128	64	32	16	8	4	2	1	0.5	0.25	0.125	0.06	0.03	≤0.015	MIC_{50} (µg/mL)	MIC_{90} (µg/mL)
PEN[e]	15	26	11	16	18	13	8	13	5	13	9	7	7	35	4	64
OXA[*]										12	35	111	34	4	0.06	0.125
AMX		2	4	12	18	15	21	25	20	27	10	13	14	15	0.5	8
AMC					1			1	14	41	71	34	22	12	0.125	0.25
CEFX	4	1		1	2	1	23	99	63			2			1	2
CAZ	6	2	4	5	8	8	86	75	2						2	8
COV	4				1	1	1	5	9	33	88	50	3	1	0.125	0.5
IPM					1			1	2	2	1	4	22	163	0.015	0.03
GEN[*]		1	9	25	7	3		1	10	27	78	29	1	5	0.125	16
TOB[+]			2	17	18	8	1		4	31	71	39		5	0.125	8
AMK	1	2			1	14	47	111	17	2		1			1	2
OTC	13	21	33	12	3	1		1	6	19	53	25	6	3	0.25	64
DOX				2	25	18	21	12	6	4	1	2	3	102	0.015	8
AZM[*]	65	7					1	2	10	54	36	17	3	1	0.25	512
RIF[*]						1	2	1	2					190	<0.015	<0.015
CLI	48	6	3	3	2		3	3	1	1	12	44	54	16	0.06	512
FFC	1			2		2	43	141	7						1	2
CHL[+]		2	52	12	1	30	93	6							2	32
CIP	2		2	6	6	1	1	3	13	13	48	61	37	3	0.06	1
ENR	1	1	3	1	5	6		8	5	10	35	68	44	9	0.06	1
MAR	1		2	12	3	2	2	9	20	52	72	14	2	5	0.25	2
PRA					1	1	5	11	6	5	3	36	105	23	0.03	0.5
SXT[*]	4	7	6	7	8	5	9	26	34	36	39	14	1		0.5	16
VAN[*]							5	28	86	70	6	1			0.5	1

Breakpoints were interpreted according to CLSI VET01S (7th edition, 2024). [*]Based on human clinical breakpoint. [e]Based on equine clinical breakpoint. [+]The canine clinical breakpoint established for gentamicin was applied to tobramycin, while the canine clinical breakpoint established for chloramphenicol was applied to florfenicol.

Supplementary Table S3. MIC distribution of antimicrobial agents against methicillin-resistant *Staphylococcus pseudintermedius* (MRSP) isolates (n = 47), including MIC_{50}, MIC_{90} values; red lines indicate clinical resistance breakpoints (CLSI VET01S, 7th edition, 2024).

Antibiotics	≥128	64	32	16	8	4	2	1	0.5	0.25	0.125	0.06	0.03	≤0.015	MIC_{50} (µg/mL)	MIC_{90} (µg/mL)
PEN[e]	19	13	4	3	2	2	4								64	256
OXA[*]	21	3	7	3			2	2	9						64	512
AMX	8	16	4	4	3	3	4	1	4						64	128
AMC	4		1	5	6	6	4	14	7						2	16
CEFX	24	6	2	4	7	4									128	256
CAZ	19	4	8	6	7		1	2							32	512
COV	28			3	2	1	7	1		5					512	>512
IPM	2	1	3	1	2	1	2	4	7	4	4	8	3	5	0.25	32
GEN[*]	2	1	11	14	11		3	1	1	1	2				16	32
TOB[+]	3	2	6	14	9	6	1	2	1	1	2				16	32
AMK	10	2		2	5	9	10	7	2						4	128
OTC	11	13	10	3	1		1		2	4	2				64	128
DOX		3	1	4	9	4	12	5	1		1			7	2	16
AZM[*]	33	2		1	7		2	1		1					256	512
RIF[*]						1	4	3	6	2			1	30	<0.015	1
CLI	30	3	2	4	4	1			1				1	1	512	>512
FFC						3	14	30							1	2
CHL[+]		2	10	3	1	17	14								4	32
CIP	4	9	9	3	2	1	4	5	3	3	1	1	1	1	16	64
ENR	8	2	4	4	8	3	3	3	3	3	3	2	1		8	256
MAR	2		9	12	5	3	5	3	1	4	2	1			8	32
PRA				1	5		13	11	8	2	1	2	4		1	8
SXT[*]	12	1	7	12	2	3	2	1	4	2	1				16	256
VAN[*]	3		1				2	7	20	13	1				0.5	2

Breakpoints were interpreted according to CLSI VET01S (7th edition, 2024). [*]Based on human clinical breakpoint. [e]Based on equine clinical breakpoint. [+]The canine clinical breakpoint established for gentamicin was applied to tobramycin, while the canine clinical breakpoint established for chloramphenicol was applied to florfenicol.

Supplementary Table S4. Clinical breakpoints used for antimicrobial susceptibility interpretation of canine *Staphylococcus pseudintermedius* isolates according to CLSI VET01S (7th edition, 2024).

Antibiotic	S	I	R
**PENe**	≤0,5	1	≥2
**OXA***	≤0,25	-	≥0,5
**AMX**	≤0,25	-	≥0,5
**AMC**	≤0,25	0,5	≥1
**CEFX**	≤2	-	≥4
**CAZ**	-	-	-
**COV**	≤0,5	1	≥2
**IPM**	-	-	-
**GEN***	≤4	8	≥16
**TOB+**	≤4	8	≥16
**AMK**	≤4	8	≥16
**OTC**	≤0,25	0,5	≥1
**DOX**	≤0,125	0,25	≥0,5
**AZM***	≤2	4	≥8
**RIF***	≤1	2	≥4
**CLI**	≤0.5	1-2	≥4
**FFC**	≤2	4	≥
**CHL+**	≤2	4	≥8
**CIP**	-	-	-
**ENR**	≤0,06	0,125-0,25	≥0,5
**MAR**	≤0,125	0,25	≥0,5
**PRA**	≤0,25	0,5-1	≥2
**SXT***	≤2	-	≥4
**VAN***	≤4	8-16	≥32

Breakpoints were interpreted according to CLSI VET01S (7th edition, 2024). [*]Based on human clinical breakpoint. [e]Based on equine clinical breakpoint. [+]The canine clinical breakpoint established for gentamicin was applied to tobramycin, while the canine clinical breakpoint established for chloramphenicol was applied to florfenicol.

Supplementary Table S5. Epidemiological cut-off values (ECOFFs) for Staphylococcus pseudintermedius provided by EUCAST, used for wild-type and non-wild-type classification.

Antibiotic	ECOFF (µg/mL)
**AMX**	0.03
**CEFX**	4
**GEN**	0.25
**OTC**	0.5
**DOX**	0.125
**CLI**	0.25
**FFC**	8
**ENR**	0.5
**SXT**	0.125

Supplementary Table S6. Oligonucleotides used for mecA and mecC gene specific polymerase chain reaction in this study.

Primer name	Primer sequence 5' – 3'	Annealing	Target	Reference
mecA-P4	TCCAGATTACAACTTCACCAGG	59	mecA complex	(34)
mecA-P7	CCACTTCATATCTTGTAACG			
mecA-F	CCTAGTAAAGCTCCGGAA	60	mecA complex	(33)
mecA-R	CTAGTCCATTCGGTCCA			
mec$_{ALGA251}$MultiFP	GAAAAAAAGGCTTAGAACGCCTC	59	mecC complex	(34)
mec$_{ALGA251}$MultiRP	GAAGATCTTTTCCGTTTTCAGC			
mecC-F	TGTTGTAGCAATGTTCACAC	55	mecC complex	(33)
mecC-R	CAAGCACTTAATATCAACGC			

Supplementary Table S7. Classification of antimicrobial agents into classes used for multidrug resistance (MDR) analysis.

**Antimicrobial class**	**Antimicrobial agents**
β-lactams	Penicillin (PEN), Oxacillin (OXA), Amoxicillin (AMX), Amoxicillin-clavulanic acid (AMC), Cephalexin (CEFX), Cefovecin (COV)
Aminoglycosides	Gentamicin (GEN), Tobramycin (TOB), Amikacin (AMK)
Tetracyclines	Oxytetracycline (OTC), Doxycycline (DOX)
Macrolides	Azithromycin (AZM)
Lincosamides	Clindamycin (CLI)
Phenicols	Florfenicol (FFC), Chloramphenicol (CHL)
Fluoroquinolones	Enrofloxacin (ENR), Marbofloxacin (MAR), Pradofloxacin (PRA)
Folate pathway inhibitors	Trimethoprim–sulfamethoxazole (SXT)
Rifamycins	Rifampicin (RIF)
Glycopeptides	Vancomycin (VAN)
